# Hospital response challenges and strategies during COVID-19 pandemic: a qualitative study

**DOI:** 10.3389/fpubh.2023.1167411

**Published:** 2023-06-30

**Authors:** Leila Mohammadinia, Vahid Saadatmand, Hassan Khaledi Sardashti, Saeid Darabi, Fahimeh Esfandiary Bayat, Nahid Rejeh, Mojtaba Vaismoradi

**Affiliations:** ^1^Department of Health Policy and Management, School of Management and Medical Informatics, Tabriz University of Medical Sciences, Tabriz, Iran; ^2^Department of Health in Disasters and Emergencies, School of Health Management and Information Sciences, Shiraz University of Medical Sciences, Shiraz, Iran; ^3^Emergency Medical Services, Fars Pre-hospital Emergency Organization, Shiraz University of Medical Sciences, Shiraz, Iran; ^4^Health Services Management, Quality Improvement Chief of Chamran Hospital, Shiraz University of Medical Sciences, Shiraz, Iran; ^5^Department of Nursing, Faculty of Nursing and Midwifery, Shahed University, Tehran, Iran; ^6^Faculty of Nursing and Health Sciences, Nord University, Bodø, Norway; ^7^Faculty of Science and Health, Charles Sturt University, Orange, NSW, Australia

**Keywords:** hospital, COVID-19 pandemic, qualitative study, healthcare, health policy

## Abstract

**Background:**

At the beginning of the COVID-19 pandemic, healthcare managers at hospitals did not have sufficient experiences to appropriately respond to the COVID-19 outbreak. Due to a lack of preparedness, many challenges arose in the healthcare system, and each country developed and implemented strategies depending on national policies. This study aimed to understand challenges during the COVID-19 pandemic and strategies used in Iranian hospitals.

**Methods:**

A qualitative research was conducted in four hospitals in an urban area of Iran. In-depth semi-structured interviews were performed with 32 participants including healthcare managers, nurses, and medical doctors. Data underwent qualitative content analysis.

**Results:**

Four categories were developed: ‘capacity expansion’, ‘management affairs’, ‘diagnostic services,” and therapeutic services’. Each category consisted of 2–3 subcategories.

**Conclusion:**

Hospitals should be prepared to intelligently respond to future epidemics. It is necessary to develop a comprehensive epidemic plan for the management of disasters to reduce the impact of the epidemic and minimize the risk to public health and ensure that resources are allocated in an efficient and effective manner.

## Introduction

The World Health Organization (WHO) declared COVID-19 as a pandemic on March 11, 2020 (1). The Iranian Ministry of Health and Medical Education (MoHME) notified in February 2020 that COVID-19 has entered the country, and that the healthcare system must be ready to deal with an unknown viral infection. From March 20, 2020, all members of the society were asked to stay at home and take precautionary measures (2). Until November 4, 2022, the number of infected people with COVID-19 in the world were 755,041,562 and 6,830,867 died (3).

Hospitals are vital infrastructures for responding to the COVID-19 pandemic, as they admit and treat patients affected by this disease (4). Furthermore, they have to deliver routine services, which doubles burden on them. Due to the limited knowledge and experience about this newly emerging disease, the COVID-19 pandemic caused many challenges to hospitals in providing healthcare services (5). Complex and expensive diagnostic-therapeutic equipment and methods, staff and hospital bed shortages, disproportionate management for the distribution of resources, lack of a standard program for preparation and response phases, inadequate staff training and practice, and different experiences of healthcare managers complicated the situation (6).

Hospital challenges for the management of the COVID-19 pandemic have been mostly related to human and logistics resources, finance and budget, psychological issues, and infection prevention (7). Various strategies were used to meet these challenges in each care context based on the national structures of countries to increase the resilience of hospitals. In Italy, structural changes included placing filter zones among different wards and ensuring the presence of airborne infection isolation rooms at least in the emergency departments. Also, technological improvements were made for the remote delivery of healthcare services. Operational measures such as assessing the risk of infection before admission, dividing acute-care from low-care assets were devised (8). The design of special pandemic hospitals in a limited period of time was one of the adaptations of the healthcare system in Australia. It was designed based on the pandemic mode with disaster capabilities having several pandemic specific features (9). In addition to coping with structural changes, process and functional reforms were used. Infection control and prevention was one of the global challenges.

Lack of personal protective equipment and infection control processes in the community and medical centers was a major global concern. The use of infection control equipment, establishment of outpatient clinics outside the hospital in order to manage symptomatic patients and isolation processes were some functional and process strategies (10). A hospital-based cross-sectional survey in Egypt, Saudi Arabia, the UAE, Qatar, Australia, the UK, and Ireland indicated the appropriate preparedness of hospitals during the COVID-19 from the perspectives of healthcare professionals (11). This qualitative study aimed to understand challenges during the COVID-19 pandemic and strategies used by healthcare professionals in Iranian hospitals.

## Methods

### Design

This qualitative research was conducted in an urban area in the south of Iran from October 11, 2020 to December 2022, using the content analysis approach. Qualitative content analysis helps with understanding social phenomena with the consideration of both manifest and latent content of data. We used the conventional content analysis approach to develop categories from data (12). The article was reported using the consolidated criteria for reporting qualitative research (COREQ; Supplementary Table 1).

### Setting and participants

Four hospitals in an urban area in south of Iran were selected for data collection. The selected hospitals admitted the COVID-19 patients on various acuity levels. Using purposive sampling, the participants were chosen. They were members of the Hospital Incident Command System (HICS) including healthcare managers, nurses, and medical doctors who had an active role in the provision of health care in the hospitals at the outset of pandemic.

Inclusion criteria were having a continuous and uninterrupted presence in the hospital at the pandemic outset in the last 2 years. Probable participants who had rich experiences of the study phenomenon were introduced through the disaster risk reduction (DRR) committee and managers of the hospitals. The list of participants’ names and their phone numbers was prepared. The main researcher contacted them regarding their interest in participation in this research. Necessary coordination for interview sessions in the time and place convenient to them was made. Due to the COVID-19 peaks and the importance of social distancing, follow-ups were performed through phone calls and the use of WhatsApp. Socio-demographic profile of the participants and hospital characteristics has been shown in Table 1.

**Table 1:** Socio-demographic profile of the participants and hospital characteristics.

Variable	Description
Age	25–57 years
Gender	18 women and 14 men
Study area	South of Iran
Institutions	Hospital A (public, general with 200 beds, center for admitting the COVID-19 patients), 11 participants (P1–P11) were interviewed.
	Hospital B (public, general and teaching with 824 beds, supporting hospital), 8 participants (P12–P19) were interviewed.
	Hospital C (public, general and teaching with 479 beds, supporting hospital), 6 participants (P20–P25) were interviewed.
	Hospital D (public, trauma center, public and teaching with 167 beds, supporting hospital), 7 participants (P26–P32) were interviewed.
Type of admission	All hospitals admitted all types of COVID-19 patients (with any level of acuity)
Occupation	6 doctors (2 general physician and 4 emergency medicine specialists)
	14 nurses (emergency room, intensive care unit (ICU) COVID-19 and the COVID-19 ward)
	12 healthcare managers (hospital command members and managers)

### Data collection

Data were collected through in-depth face-to-face semi-structured interviews using a pilot-tested interview guide by the first author (VS). The interviews began with an open-ended question: Can you tell me about your experiences of the COVID-19 pandemic? The interview topic was then shifted to the research objectives and the interviewee’s more specialized field of experience dealing with COVID-19 as follows: What were the problems and challenges in your ward in the COVID-19 pandemic? What strategies did you use to meet the challenges?

The interview locations were tailored to the interviewee’s comfort in a peaceful setting where the participants felt comfortable as were mainly staff rooms in the ward. The average interview duration was 45 min. All the participants were interviewed only once during the third and fifth waves of COVID-19. The data gathering was continued until data saturation was reached and the researcher received redundancy signals that data collection could be ceased.

### Data analysis

The data collection and data analysis were performed simultaneously using qualitative content analysis. After transcribing each interview, coding was performed, and then the subsequent interview was conducted. The conversational dialogue within the interviews was converted into writing. To apprehend the essential features of data and to obtain the sense of whole, the researchers read the transcripts several times. In addition, field notes along with the interview data helped improve our understanding of the study phenomenon. Considering the consistency of the coding process among different coders/analysts, the coding process performed and supervised by the research team.

The leader of the research team employed two experienced people to code the transcripts. Several teaching and training sessions were held to ensure of reliability in coding. Training included a pilot checking stage where two people applied codes to sample data and then discussed their work to recognize areas of agreement and discrepancy in coding. Discussions led the research team to clarify and amend the coding design and the coding process. In the open coding stage, 748 initial codes were extracted. Initial codes were compared together based on their similarities and differences and were classified based on their relevance. Categories were developed and were labeled, so that the internal stability of each category and its differences with other categories were maintained (13). MAXQDA software was used for data management.

### Rigor

To ensure rigor, long-term engagement with the participants, peer and member checking, and presentation of a rich description of data collection and data analysis were used (14). In addition to continuous relation with the participants, the research team persisted the study for a long period and spent enough time for data collection and participants’ follow-up. For peer checking, the transcriptions along with coding and initial categories were shown to two qualitative researchers. Their confirmation was achieved after incorporating their comments. For member checking, a brief report of the interviews and extracted codes were presented to the participants who confirmed it. The researcher clarified aspects in the participants’ accounts that were ambiguous through phone calls and WhatsApp. For transferability, attempts were made to consider maximum variation in sampling in terms of work experience and work position in the hospitals.

### Ethical considerations

This study was approved by Shiraz University of Medical Sciences (decree code: IR.SUMS.REC.1400.125). The principle of confidentiality of data and audio-recorded files was considered with care. During the interviews, social distancing and infection prevention protocols were adhered. Prior to the interviews, all participants were briefed about the objectives and method of the study and then were requested to provide written informed consent. The participants were free to withdraw from the study at any time.

## Results

The study participants were 32 people consisting of 18 women and 14 men aged 22–57 years with a work experience of 2–29 years. They were 2 medical doctors, 16 clinical nurses, and 14 healthcare managers. They had a level of education ranging from bachelor’s degree to medical doctor. Socio-demographic profile of the participants and hospital characteristics have been shown in Table 1.

### Interview results

The data analysis led to the development of four categories as follows: ‘capacity expansion’, ‘management affairs’, ‘diagnostic services’, and therapeutic services’ (Table 2). Each category consisted of 2–3 subcategories. After the description of each category, the subcategories were explained and supported with sample quotations from the participants. The presented quotes were most illustrative, insightful, or representative of the subcategories or patterns identified in the data analysis, and illustrated key findings for a richer understanding of the research topic.

**Table 2:** Main categories and sub-categories developed in the study.

Main category	Sub-category	Participants
Capacity expansion	Physical space	Participant1,P4,P5,P7,P8,P13,P14,P16,P19,P27,P31
	Human resources	P1,P2,P4,P5,P6,P11,P13,P14,P15,P17,P26
	Equipment	P1,P5,P6,P10,P11,P15,P19,P26,P28,P29
Management affairs	Comprehensive epidemic program	P7,P9,P11,P13,P18,P19,P21
	Finance and support	P1,P6,P8,P9,P11,P20,P32
	Information services	P4,P8,P21,P25,P26
Diagnostic services	Laboratory test	P1,P3,P4,P7,P22
	Diagnostic imaging	P1,P3,P4,P6,P13
Therapeutic services	Screening	P1,P2,P3,P4,P6,P7,P22
	Clinical care	P1,P2,P4,P6,P8,P11,P16
	Infection prevention and control	P3,P5,P8,P16,P21

### Capacity expansion

It was one of the main concerns by healthcare authorities in the COVID-19 pandemic. In addition to routine services delivery, the healthcare systems had to improve the medical surge capacity as to respond to markedly increased volume of patients and the high load of infectious and critically ill patients. For this purpose, the hospitals should have expanded their physical space, human resource, and equipment.

#### Physical space

Given the lack of standard hospitals for managing the emerging pandemic, structural and non-structural challenges were identified. They were lack of physical space and beds, special part of patient’s entry and exit, inadequate ventilation, and insufficient installation systems. These problems were caused due to the nature of the hospitals that were mostly general and were not specially designed to manage health disasters and pandemics.

“We had to turn non-clinical spaces into COVID-19 wards; for example, the cardiac care unit (CCU) was transformed into a COVID-19 intensive care unit (ICU), which was very challenging” (Participant 1, ICU Head nurse).

Several strategies were used to solve these challenges. Increasing the hospital’s physical capacity included allocating some supporting hospitals during peak times and repurposing existing wards for COVID-19 departments alongside non-structural changes. It encompassed creating a physical space and setting up an outpatient clinic to prevent overcrowding of hospitals by COVID-19 patients. Hospitals were classified to A, B, C, and D and were supported by clinics located out of the central building. The outpatient clinics admitted non critically COVID-19 patients, who received some diagnostic and therapeutic interventions including radiology services, laboratory tests, antiviral medications, and fallow-ups. The function of the outpatient clinic was such that the patients of Covid-19 who needed outpatient medical services would file a case at the hospitals and then referred to the clinics to receive intravenous antiviral drugs. These centers were operated in two work shifts in a daily manner.

“Some other departments were allocated to COVID-19. To increase the capacity of ICU department, they changed the cardiac care unit (CCU) to the COVID-19 ICU because its structure was more similar to an ICU, and less structural changes were required” (Participant 16, CCU Head nurse).

**Figure 1:**
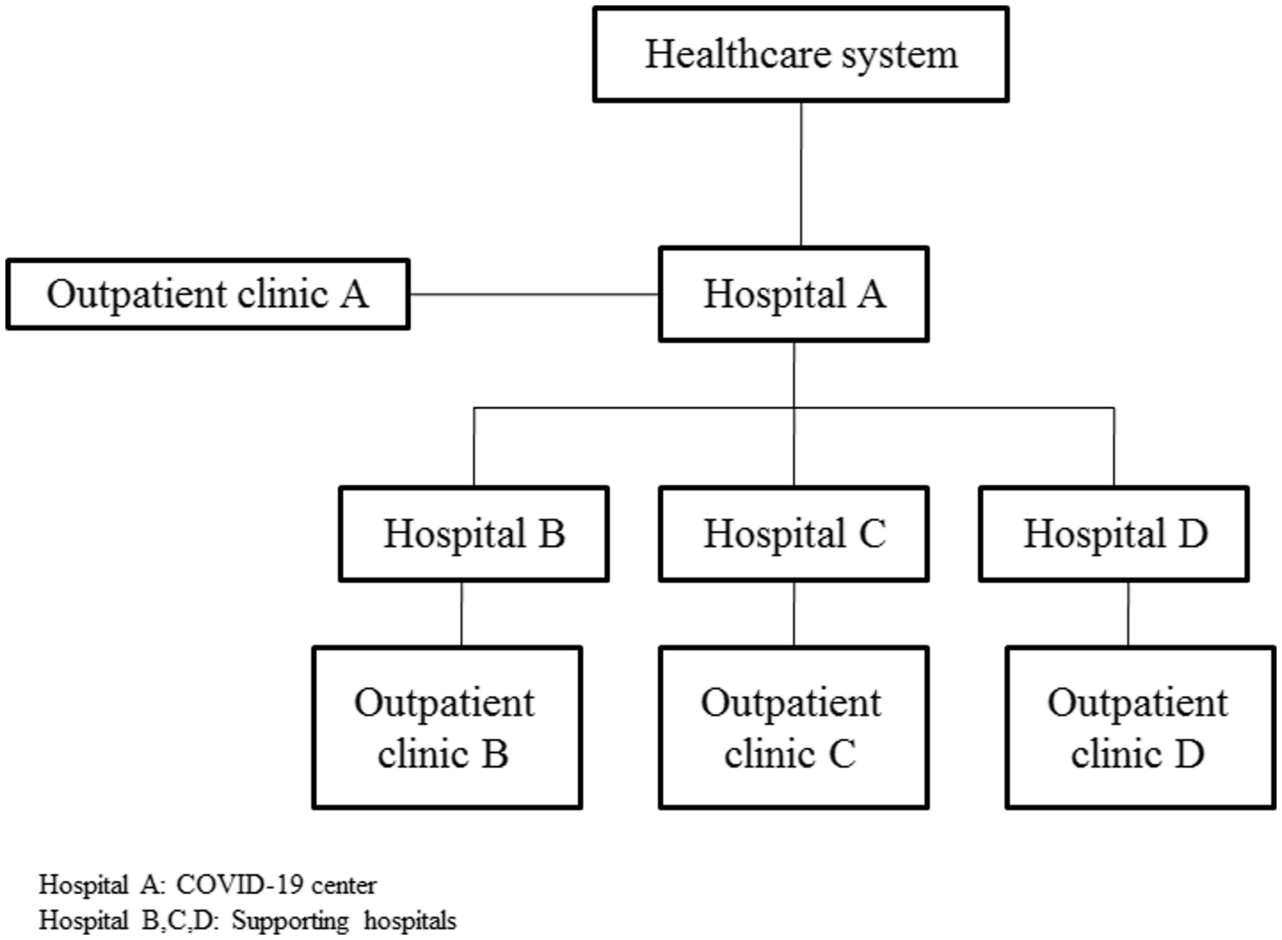
Hospitals stratification during the COVID-19 pandemic.

Hospitals were allocated in such a way that hospital A was considered the center and based on the prediction of epidemiologists and during peak times, excess COVID-19 patients were referred to hospital B, C, and D, respectively. During peaks, hospitals A, B, C and D were equipped with supplies to care for COVID-19 patients at any level of acuity. After that the peaks subsided, supporting hospitals (B, C, and D) returned to their common status and delivered their routine services. Figure 1 depicts the hospitals stratification during the COVID-19 epidemic.

“Although our hospital was a support center, we admitted the COVID-19 patient with any level of disease acuity. For this purpose, we had to be equipped with special supplies” (Participant 16, CCU Head nurse).

#### Human resources

The most crucial factor influencing the management of the COVID-19 pandemic was human resources. Staff shortages, physical and mental health problems and empowerment issues were emphasized. Even though the hospital managers mentioned staff shortages as the most important issues, according to the nurses and doctors, heavy workloads and physical and mental problems were more annoying. All hospitals reported this challenge, which multiplied the burden on healthcare staff as well as reduced the quality of care. Staff shortages and empowerment issues were more tangible in supporting hospitals (B, C, and D), because they had to change their services frequently during and between pandemic peaks.

“Due to the lack of man power, more patients were divided among us, and this caused problems and challenges in the provision of healthcare” (Participant 4, Nurse).

Strategies used to provide manpower were mostly situational and were mainly taken at times of peaks. During the peaks, mostly due to the closure of some departments or the cancellation of elective surgeries, the extra staff of closed departments were summoned to work in the COVID-19 department. In addition, the use of contract employees, volunteers, military staff, and retired personnel was an effective strategy to reduce the impact of staff shortages.

“Another good experience concerning the provision of manpower was the use of volunteers; they mostly might not have performed some special operations, but supported medical staff in the hospital” (Participant17, COVID-19 Head nurse).

Almost all participants acknowledged the presence of physical and mental problems among healthcare staff. At the beginning of the pandemic, due to the psychological atmosphere of the society, the staff experienced social, family and professional disturbances. COVID-19 infection, hard working conditions, difficulty in the use of personal protection equipment (PPE), and social or family concerns affected their physical and mental health.

“In addition to fear, anxiety, fatigue, and depression, physical symptoms such as headaches and palpitations persisted in some staff for a long time” (Participant 5, Nurse).

Motivational factors in terms of material, spiritual, social and familial, and rotating work shifts caused physical and mental pressures. They needed mental-psychological support by psychologists and psychiatrists to relieve their symptoms.

“I believe anyone who wants to work in the COVID-19 department must have strong and high spirit, motivation, humanity, and conscience. Humanity has no boundaries” (Participant 11, Nurse).

Empowering personnel during the COVID -19 pandemic was a difficult task. First, because of social distancing, effective education was impossible. Also, when adequate external workforce and staff were needed, volunteers with different educational levels and different groups had to be trained. Personal protection against infection and the management of critically ill patients were main concerns at hospitals. The complex conditions of COVID-19 patients, and lack of knowledge, experience and skills indicted the need for staff education and training.

“Staff had not received training to care for critically ill COVID-19 patients. They did not know how to care for such patients” (Participant 6, Educational supervisor).

Training teams were developed to manage staff training through peer training, specialized teams training, and via special offline guidelines and tele-methods. Developing a training package consisted of virtual and face-to-face methods for the acquisition of experience and specific clinical skills.

“Telecommunication methods must be combined with face-to-face methods and the staff should acquire skills and experiences” (Participant 6, Educational supervisor).

#### Equipment

Oxygen supply and provision of equipment for the monitoring of ventilation and oxygenation were crucial concerns in the hospitals, especially in the early days of the outbreak. The lack of respiratory equipment, PPE, and medications during COVID-19 peaks, and the enhanced hospitalization rate posed many challenges. To overcome the challenge for medication supply and other equipment, the capacities of the private sector, non governmental organizations (NGOs) and charities, and organizations such as the Red Crescent and army were required. The use of up-to-date technologies such as oxygen generator devices to supply oxygen needed by the hospitals largely solved this problem.

“During COVID-19 peaks, the hospital faced equipment, medication and oxygen shortages. Ventilation equipment including ventilators, oxygen cylinders, and oxygen generator devices were donated” (Participant 26, Manager).

### Management affairs

Most managers had no desire and positive attitude toward developing comprehensive programs in the disaster area. Targeted planning and investment in epidemics played an essential role in creating coordination and preparedness, and effective management of COVID-19.

#### Comprehensive epidemic program

The hospitals were not prepared or had no plan to respond to epidemics. Most decisions were made and implemented on a case-by-case basis. Due to the long duration of the pandemic, any experience was gained gradually, and relevant information was documented, which was used in the next peaks.

“Over time, experiences were gained whose information was documented, and we responded better in the next peaks” (Participant 13, Manager).

With the passage of time and identification of possible risks and vulnerabilities of the healthcare system, short-term and medium-term contingency plans were developed. The participants focused on engaging experts in planning, using extensive operational maneuvers, improving coordination, and recognizing system vulnerabilities. After developing this plan, the epidemic program should be practiced at the local and national level using different scenarios. Next, based on the evaluation of results, the program should be revised and essential amendments should be made. It was very important to carry out this process continuously to be adequately prepared.

“Stabilization requires comprehensive planning with the presence of experts and elites” (Participant 11, Nurse).

#### Finance and support

The hospitals’ specific revenues fell sharply due to the reduction of patients’ visits and the fear of COVID-19, cancellation of elective surgeries, and the high cost of caring for COVID-19 patients. In addition, economic and political sanctions put financial pressure on healthcare systems. Governmental budgets belonging to the states were reduced. The government dedicated a budget funding line to medical colleges called the COVID-19 funding. This budget line was financed from the country’s specific income sources. In addition to governmental budget allocation, external systems would amend financial problems indirectly. Use of the capacity of benefactors, NGOs and charities, Red Crescent, and military forces covered the gap for hospital support. The idea arose through the massive mobilization of representatives of the country’s leaders and political authorities to play a pivotal role in financial resilience and support of hospitals involved in COVID-19 pandemic.

“One of the effective measures for financial promotion and support was the attraction of charity. Donors were either volunteer” (Participant 20, Manager).

#### Information services

Almost all structural components of hospitals had their own information system where all related information was recorded. The nursing information system, imaging, administrative, financial, and laboratory were the most important information systems. During the Covid-19 pandemic, almost all units’ information systems were involved. Up-to-date information technologies played an essential role in preparedness and response processes, including coordination, training, management, early warning, information, and communication. The absence of a comprehensive and integrated system for recording and managing data related to COVID-19 conditions led to irregularities, and wasted staff time and energy. Some units had to write statistical information manually and record it electronically again. Therefore, many tasks were performed in parallel. On the other hand, each unit had its own separate information system and data was entered in duplicate. This problem caused informational and statistical confusion between the units. The suggested solution was to record all information related to COVID-19 patients in the Health Information System (HIS) and connect it to unique information center.

“Electronic methods should replace these methods, and all patient information should be recorded in a single central system. It can prevent many parallel tasks and wasting of time and energy” (Participant 4, Nurse).

### Diagnostic services

The similarity of clinical symptoms to other respiratory diseases made the isolation of COVID-19 patients a challenge. After triage, the specialized differentiation of COVID-19 patients was performed on two bases as the polymerase chain reaction (PCR) test and chest imaging.

#### Laboratory test

Challenges with PCR tests were results’ inaccuracy especially with rapid tests and delay in reporting results. The rapid PCR test was mostly unreliable. Most patients with the typical symptoms of COVID-19 had a negative rapid PCR test, while the original test was positive after 24 h. False negative results affected the treatment course.

“Patients whose rapid tests were negative had positive PCR test results. The PCR result was ready with a delay, and the suspected patient remained undecided” (Participant 7, Manager).

#### Diagnostic imaging

Chest radiography was the only method for screening suspicious cases before the PCR test. However, the inconsistencies of the CT scan report by specialists and the overuse of this method was problematic. A chest X-ray was taken from almost all patients with respiratory symptoms. In addition to the disadvantages of this method, the burden of radiology units increased. On the other hand, the risk of transmission of the virus enhanced in hospital wards, which was worrying. It caused a lot of concerns about outbreaks inside the hospital and the best solution was to appoint infectious disease specialists in the work shift or on-call as the focal point of COVID-19. These specialists assigned one person for COVID-19 patients for hospitalization, referral to outpatient clinic, or discharge from the hospital.

“Due to diagnostic contradictions and different opinions of doctors, we had to appoint a person as the focal point of COVID-19 to determine the assignment of suspected respiratory patients in the gray zone” (Participant 13, Nurse).

### Therapeutic services

During the pandemic, the treatment guidelines for the COVID-19 patients were variable. Although all medical centers followed the CDC and WHO guidelines for the COVID-19 management, these protocols needed to be localized according to local resources and facilities. Sometimes, there was no access to some resources, including special drugs and advanced equipment. Therefore, due to limited resources medical centers had to use local protocols.

#### Screening

Because of the similarity of COVID-19 symptoms to other respiratory diseases such as asthma, chronic obstructive pulmonary disease (COPD), influenza, etc., the diagnosis and isolation of suspected cases caused confusion. At the beginning of the pandemic, all patients with respiratory problems had to take a PCR test. This process was not only expensive, but also caused the hospital congestion. Therefore, it was important to screen the COVID-19 patients exactly.

A two-layer triage where respiratory patients were triaged in two separate stations was the strategy. The first layer was located near the emergency department based on hospital structure. In this unit, a staff nurse screened the suspicious patients with respiratory symptoms based on triage checklist. Next, those patients were directed to another room in emergency department called ‘gray zone’. This zone was a temporary department, which was the waiting area for suspicious patients to identify COVID-19 cases. In addition to primary observation and intervention, chest X-ray and rapid PCR test were taken in the gray zone. After that the test results were determined, patients in the gray zone were transferred to the COVID-19 department or discharged from the COVID-19 services.

“Those patients who come here were checked for vital signs and possible symptoms of the coronavirus. If anyone was suspicious, they entered a section called the gray zone at the next station. If the patient’s oxygen saturation was low, a chest X-ray was taken. It effectively distinguished COVID-19 from non-COVID-19 respiratory patients” (Participant 3, Emergency supervisor).

#### Clinical care

Insufficient knowledge about COVID-19, different treatment protocols and rapid changes caused ambiguities and doubts among healthcare staff. Due to the lack of some therapeutic device or medications, clinical decisions were overshadowed. For example, during a period of the pandemic, the treatment guideline was to intubate COVID-19 patients with delay. Due to the lack of advanced equipment such as non-invasive ventilation devices, it was impossible to follow this protocol. The positive caring experiences of healthcare staff improved the clinical condition of COVID-19 patients. Although they had not faced the epidemics in the past, they became experienced over time and devised strategies such as noninvasive ventilation, prone positioning to improve oxygenation, supportive care methods, communication skills, and advocating specialized respiratory teams. The respiratory teams that were independent from the CPR (cardiopulmonary resuscitation) team were developed from selected critical care personnel who received advanced training in the provision of care for COVID-19 patients.

“Supportive measures and nursing care had the greatest effect on the healing and recovery of COVID-19 patients than following up medication regimens and antiviral protocols, etc” (Participant 11, Nurse).

#### Infection prevention and control

Being infected by COVID-19 was the first concern of the healthcare managers. It caused the loss of staff and affected the provision of healthcare services. Infection prevention and control played a significant role to avoid this challenge. Structural problems of hospitals and lack of infection control equipment were also mentioned. Another concern during COVID-19 was the presence of in-hospital outbreak. Medical centers did not have structural standards related to infection control. COVID-19 wards were not isolated and the COVID-19 patients were not tracked properly. In COVID-19 hospitals, the isolation of COVID-19 structures and equipment were not well observed and only administrative wards and management departments were isolated. This caused a significant challenge in supporting hospitals because infection could be transmitted to other wards, which provided routine care.

“There are a lot of common pathways and equipment. Surgical patients were sent to the radiology department for CT scan, which was the same for COVID-19 patients and this might contaminate the environment” (Participant 8, Nurse).

Since the supporting hospitals did not meet the standards for epidemic conditions, all equipment used for COVID-19 patients were isolated. This action was carried out through non-structural changes of departments, entry and exits, and process modifications.

“The special equipment related to COVID-19 was separated as much as possible, including x-ray and ultrasound diagnostic equipment, and they were allocated as portable devices for COVID-19 patients” (Participant 8, Nurse).

## Discussion

This study established the ground for assessing, identifying, and preparing for an effective response to the emerging and re-emerging COVID-19 pandemic. A challenge of the hospitals in the COVID-19 pandemic was to increase the hospital medical surge capacity. Lack of a hospital with special features forced hospital managers to admit COVID-19 patients into general hospitals leading to concerns about in-hospital outbreaks. The stratification of hospitals and the establishment of outpatient clinics made that a hospital was considered a COVID-19 center and played as the first layer of accepting COVID-19 patients. Supporting hospitals accepted COVID-19 patients as the next layers if a hospital reached its total capacity. The structure of wards should be standard for the emerging disease. Any increase in the physical capacity of the hospital requires supplies, equipment, and medication in the early days of the outbreak (10, 15). Hospital preparedness requires the transformation of infrastructures and capacities in response to changing situations and at the same time the provision of normal patient care (16).

The hospitals counted on other organizations for help including charities, NGOs, the military, and the Red Crescent. One of the milestones for the identification of developing and underdeveloped healthcare systems is the mobilization of the domestic public and the support of external and international organizations (6, 7, 17). The shortage of workforces leads to an increase in staff workloads (18– 20). The use of contract employees, retirees, military forces, and invitation of volunteer forces are some strategies to temporarily resolve this problem (18, 19). The difficult work condition during the COVID-19 pandemic in terms of exhausting PPE causes numerous physical and psychological challenges that reduces resilience among healthcare staff (4, 20–22).

The formation of peer training teams for the training of new staff using special training packages was one of the strategies mentioned by the participants. The new workforce needs training, practice, and acquisition of skills and experience, which requires time, cost, and planning (23, 24). All hospitals must have a comprehensive plan to deal with disasters and emergencies. In our study, hospitals did not have a preparedness plan to respond to the emerging pandemic. The experiences obtained during the COVID-19 peaks were collected by our participants and were documented to be used in dealing with the next peaks. As soon as the pandemic begins, most measures are taken contingently. Although a small number of participants benefits from the experiences of previous pandemics such as SARS and H1N1 conditions, the situation during the COVID-19 pandemic is unexpected and unpredictable (25–27). Due to the unknown nature of the disease, especially in the early days of the outbreak, there are many ambiguities and challenges at all levels of treatment and care in hospitals (28, 29).

A two-layer triage and determining intermediate stations such as the gray zone to identify suspected patients for greatly reducing triage problems were mentioned. According to the current global information, uncertainties affect the appropriate response to treatment operations. Triage is the first line of contact with COVID**-**19 patients. Due to the similarity of COVID-19 symptoms to other respiratory diseases, its diagnosis is challenging, and there is the possibility of making errors such as over triage (10, 30, 31). The lack of knowledge on the respiratory system support and medications as well as care modalities are main challenges to manage the pandemic. Due to the emerging disease, the implementation protocols are accompanied by doubts, which affect nursing interventions (32, 33). These protocols should be localized first for each region, and the nursing groups should be instructed and trained continuously by specialized nursing and medical teams (34, 35).

The financial issues of hospitals due to the closure of operating rooms following the cancellation of surgeries and reduction of patient visits due to the fears of COVID-19 were reported by the participants. External organizations and charities were mentioned as key factors in the logistical support of the health system during epidemics in the hospitals. One of the most important factors in hospital resilience is logistical support (36, 37). Although ministries and governments are the main authorities of healthcare support during the pandemic, lack of support by NGOs and social organizations with a coherent and coordinated program is evident in other healthcare systems (25, 38).

Another challenge according to our study was diagnostic methods as delay in notifying the results of the test and inaccuracies in results. Non-structural changes and modification of isolation processes and management of protective personnel requirements in supporting hospitals, as well as the local arrangements of the hospital were some solutions. In the early days of the outbreak, due to the lack of laboratory kits, the only method to diagnose suspected cases was a chest CT scan accompanied by conflicting specialist reports, access restriction, and a lack of separate CT equipment for suspected COVID-19 patients (39, 40). Despite these diagnostic problems, the participants mentioned the decisive role of infectious disease specialists as a focal point in the assignment of suspicious cases. The majority of components, structures and equipment are used for COVID-19 as well as other patients, which increases the risk of infection transmission. Lack of PPE and disinfection equipment, especially in the early days of the pandemic, has been a common challenge faced by hospitals (10, 41, 42).

The lack of an integrated information system between healthcare departments was this study finding. It has been often acknowledged that existing systems including HIS have many shortcomings and other systems have no procedural unity (8, 43, 44). The proposed solution is to transfer all information related to the COVID-19 patient to the HIS and connect it to unique information center.

The most successful experiences in the COVID-19 pandemic was the national mobilization, which was implemented through a national summon. All the Iranian people participated in this call in different ways. The most important functional area of this call is in the provision of resources including hospital staff and equipment. Certainly, this plan is associated with problems such as lack of coordination, disproportionate distribution of resources, and organizational integrity. Therefore, to implement the national mobilization plan, it is suggested to establish a specific system for epidemics to link and integrate government, private and non-governmental systems.

### Limitations

A relatively large sample size comprising different healthcare groups was recruited to collect rich and diverse data, but difficulty in accessing managers and risk of infection transmission might have affected the quality of data collection. In addition, some individual problems, including heavy workloads, fatigue, and limited free time made the staff unwilling to participate in the study. Due to busy work, only six doctors agreed to be interviewed and it might disturb sample balance.

## Conclusion

Major challenges faced by healthcare managers and staff in hospitals in response to the COVID-19 pandemic were described along with main strategies used to meet these challenge. It is necessary to develop a comprehensive epidemic plan for the management of disasters to reduce the impact of the epidemic and minimize the risk to public health and ensure that resources are allocated in an efficient and effective manner.

Several items including the healthcare capacity expansion, optimization of management function, attracting financial support, improvement of diagnostic and therapeutic services in epidemics, and integration of health information systems should be considered in developing the comprehensive program during the pandemic. This program should be practiced at the local and national level using different scenarios to increase healthcare readiness to effectively respond to future pandemics.

The healthcare capacity is crucial in preparing for future pandemics. Sufficient resources, including healthcare facilities, equipment, and personnel should be provided to cope with increased demand during a pandemic. It encompasses investing in infrastructure, workforce training, and resource allocation to enhance healthcare capacity and resilience. Moreover, management functions during a pandemic in terms of effective coordination, communication, and decision-making within healthcare systems and among various stakeholders should be improved. Robust management strategies, protocols, and governance structures for efficiently responding to and managing future pandemics are required. Increased investments in public health infrastructure, research and development, and healthcare systems, as well as exploring innovative financing mechanisms to ensure sustained financial support during future pandemics are emphasized. There is a need for readily available, accurate, and efficient diagnostic tools and treatment modalities and preventive measures to improve patient care and outcomes during pandemics. Interoperable and connected systems for efficient data sharing, surveillance, monitoring, and response coordination for standardized data collection and reporting mechanisms, as well as fostering collaboration between public health agencies and healthcare providers should be devised.

Future research should follow up and identify the effectiveness of strategies and measures used during COVID-19 pandemic and use gained experiences for efficient responses to future pandemics.

## Data availability statement

The anonymized raw data supporting the conclusions of this article will be made available by the authors, without undue reservation.

## Ethics statement

The studies involving human participants were reviewed and approved by Shiraz University of Medical Sciences (decree code: IR.SUMS.REC.1400.125). The participants provided their written informed consent before the data collection.

## Author contributions

LM and VS: conceptualization and methodology. VS, SD, and FEB: investigation and formal analysis. LM, VS, SD, HKS, and FEB: writing—original draft preparation. LM, VS, HKS, NR, and MV: writing—review and editing. All authors have read and approved the final manuscript and agreed to be accountable for all aspects of the work in ensuring that questions related to the accuracy or integrity of any part of the work are appropriately investigated and resolved.

## Funding

This study was approved by Shiraz University of Medical Sciences, Iran (code IR.SUMS.REC.1400.125).

## Conflict of interest

The authors declare that the research was conducted in the absence of any commercial or financial relationships that could be construed as a potential conflict of interest.

## Publisher’s note

All claims expressed in this article are solely those of the authors and do not necessarily represent those of their affiliated organizations, or those of the publisher, the editors and the reviewers. Any product that may be evaluated in this article, or claim that may be made by its manufacturer, is not guaranteed or endorsed by the publisher.
