# Supplementary material for: Hospital response challenges and strategies during COVID-19 pandemic: a qualitative study
Source: Front Public Health. 2023 Jun 30;11:1167411. doi: 10.3389/fpubh.2023.1167411 (PMC10349376; doi:10.3389/fpubh.2023.1167411)
Supplement: Supplementary file 1 [file Table_1.DOC]

**Manuscript:** **Hospital Response Challenges and Strategies during COVID-19 Pandemic: A Qualitative Study**

**Supplementary Table 1** Consolidated criteria for reporting qualitative studies (COREQ): 32-item checklist

| **No. Item** | **Guide questions/description** | **Reported on Page #** |
| --- | --- | --- |
| **Domain 1: Research team and reﬂexivity** |  |  |
| *Personal Characteristics* |  |  |
| 1. Inter viewer/facilitator | Which author/s conducted the inter view or focus group? | Vahid Saadatmand, Saeid Darabi, Fahimeh Esfandiary Bayat |
| 2. Credentials | What were the researcher’s credentials? E.g. PhD, MD | PhD, PhD candidate, MSc |
| 3. Occupation | What was their occupation at the time of the study? | Faculty Member(4), EMS staff(2), Hospital Manager(1) |
| 4. Gender | Was the researcher male or female? | They are four male and three Female |
| 5. Experience and training | What experience or training did the researcher have? | Qualitative research methodology, software education and training (MAXQDA), interview method, communication skills |
| *Relationship with participants* |  |  |
| 6. Relationship established | Was a relationship established prior to study commencement? | Yes |
| 7. Participant knowledge of the interviewer | What did the participants know about the researcher? e.g. personal goals, reasons for doing the research | On the first occasion with the participants, we explained the objectives and the necessity of this research. |
| 8. Interviewer characteristics | What characteristics were reported about the interviewer/facilitator? e.g. Bias, assumptions, reasons and interests in the research topic | The work experience of the interviewers was the most important characteristics that might have influenced the interviews. However, bracketing was performed. |

**Supplementary Table 1** Consolidated criteria for reporting qualitative studies (COREQ): 32-item checklist (continue)

| **Domain 2: study design** |  |  |
| --- | --- | --- |
| *Theoretical framework* |  |  |
| 9. Methodological orientation and Theory | What methodological orientation was stated to underpin the study? e.g. grounded theory, discourse analysis, ethnography, phenomenology, content analysis | Content analysis |
| *Participant selection* |  |  |
| 10. Sampling | How were participants selected? e.g. purposive, convenience, consecutive, snowball | Purposive sampling |
| 11. Method of approach | How were participants approached? e.g. face-to-face, telephone, mail, email | All methods were face-to-face, but due to social distancing, telephone call and virtual methods (whatsapp) were also used. |
| 12. Sample size | How many participants were in the study? | 32 participants |
| 13. Non-participation | How many people refused to participate or dropped out? Reasons? | Because of the hospital overcrowding, two emergency department supervisors withdrew from the interview. |
| *Setting* |  |  |
| 14. Setting of data collection | Where was the data collected? e.g. home, clinic, workplace | Hospital |
| 15. Presence of non-participants | Was anyone else present besides the participants and researchers? | None |

**Supplementary Table 1** Consolidated criteria for reporting qualitative studies (COREQ): 32-item checklist (continue)

| 16. Description of sample | What are the important characteristics of the sample? e.g. demographic data, date | | Experience and dealing with COVID-19 situation, member of the hospital disaster committee |
| --- | --- | --- | --- |
| *Data collection* |  | |  |
| 17. Interview guide | Were questions, prompts, guides provided by the authors? Was it pilot tested? | | Data were collected through in-depth semi-structured interviews using a pilot-tested interview guide by the first author. |
| 18. Repeat interviews | Were repeat inter views carried out? If yes, how many? | | No |
|  | |  |  |
| 19. Audio/visual recording | Did the research use audio or visual recording to collect the data? | | The voice recorder of the android system on the mobile phone was used. |
| 20. Field notes | Were ﬁeld notes made during and/or after the interview or focus group? | | Yes |
| 21. Duration | What was the duration of the interviews or focus group? | | 45 minutes |
| 22. Data saturation | Was data saturation discussed? | | Data saturation was discussed virtually by the research team in several sessions. |
| 23. Transcripts returned | Were transcripts returned to participants for comment and/or correction? | | n/a |
| **Domain 3: analysis and ﬁndings** |  | |  |
| *Data analysis* |  | |  |
| 24. Number of data coders | How many data coders coded the data? | | 748 |

**Supplementary Table 1** Consolidated criteria for reporting qualitative studies (COREQ): 32-item checklist (continue)

| 25. Description of the coding tree | Did authors provide a description of the coding tree? | They provided a description of the coding tree. |
| --- | --- | --- |
| 26. Derivation of themes | Were themes identiﬁed in advance or derived from the data? | The themes derived from the data. |
| 27. Software | What software, if applicable, was used to manage the data? | MAXQDA software. |
| 28. Participant checking | Did participants provide feedback on the ﬁndings? | Yes |
| *Reporting* |  |  |
| 29. Quotations presented | Were participant quotations presented to illustrate the themes/ﬁndings? Was each quotation identiﬁed? e.g. participant number | The participant quotations were presented in the result section. |
| 30. Data and ﬁndings consistent | Was there consistency between the data presented and the ﬁndings? | Yes, there was. |
| 31. Clarity of major themes | Were major themes clearly presented in the ﬁndings? | Yes, they were. |
| 32. Clarity of minor themes | Is there a description of diverse cases or discussion of minor themes? | Yes. It is described in the result and discussion section of the manuscript. |
